# Association of Gender With Learner Assessment in Graduate Medical Education

**DOI:** 10.1001/jamanetworkopen.2020.10888

**Published:** 2020-07-16

**Authors:** Robin Klein, Nneka N. Ufere, Sowmya R. Rao, Jennifer Koch, Anna Volerman, Erin D. Snyder, Sarah Schaeffer, Vanessa Thompson, Ana Sofia Warner, Katherine A. Julian, Kerri Palamara

**Affiliations:** 1 Division of General Internal Medicine and Geriatrics, Department of Internal Medicine, Emory University School of Medicine, Atlanta, Georgia; 2 Division of Gastroenterology, Department of Medicine, Massachusetts General Hospital, Boston; 3 Massachusetts General Hospital Biostatistics Center, Boston, Massachusetts; 4 Department of Global Health, Boston University School of Public Health, Boston, Massachusetts; 5 Department of Medicine, University of Louisville, Louisville, Kentucky; 6 Department of Medicine, University of Chicago, Chicago, Illinois; 7 Department of Pediatrics, University of Chicago, Chicago, Illinois; 8 Division of General Internal Medicine, Department of Medicine, University of Alabama at Birmingham School of Medicine; 9 Division of Hospital Medicine, Department of Medicine, University of California, San Francisco; 10 Division of General Internal Medicine, Department of Medicine, University of California, San Francisco; 11 Division of General Internal Medicine, Department of Medicine, Massachusetts General Hospital, Boston

## Abstract

**Importance:**

Gender bias may affect assessment in competency-based medical education.

**Objective:**

To evaluate the association of gender with assessment of internal medicine residents.

**Design, Setting, and Participants:**

This multisite, retrospective, cross-sectional study included 6 internal medicine residency programs in the United States. Data were collected from July 1, 2016, to June 30, 2017, and analyzed from June 7 to November 6, 2019.

**Exposures:**

Faculty assessments of resident performance during general medicine inpatient rotations.

**Main Outcomes and Measures:**

Standardized scores were calculated based on rating distributions for the Accreditation Council for Graduate Medical Education’s core competencies and internal medicine Milestones at each site. Standardized scores are expressed as SDs from the mean. The interaction of gender and postgraduate year (PGY) with standardized scores was assessed, adjusting for site, time of year, resident In-Training Examination percentile rank, and faculty rank and specialty.

**Results:**

Data included 3600 evaluations for 703 residents (387 male [55.0%]) by 605 faculty (318 male [52.6%]). Interaction between resident gender and PGY was significant in 6 core competencies. In PGY2, female residents scored significantly higher than male residents in 4 of 6 competencies, including patient care (mean standardized score [SE], 0.10 [0.04] vs 0.22 [0.05]; *P* = .04), systems-based practice (mean standardized score [SE], −0.06 [0.05] vs 0.13 [0.05]; *P* = .003), professionalism (mean standardized score [SE], −0.04 [0.06] vs 0.21 [0.06]; *P* = .001), and interpersonal and communication skills (mean standardized score [SE], 0.06 [0.05] vs 0.32 [0.06]; *P* < .001). In PGY3, male residents scored significantly higher than female patients in 5 of 6 competencies, including patient care (mean standardized score [SE], 0.47 [0.05] vs 0.32 [0.05]; *P* = .03), medical knowledge (mean standardized score [SE], 0.47 [0.05] vs 0.24 [0.06]; *P* = .003), systems-based practice (mean standardized score [SE], 0.30 [0.05] vs 0.12 [0.06]; *P* = .02), practice-based learning (mean standardized score [SE], 0.39 [0.05] vs 0.16 [0.06]; *P* = .004), and professionalism (mean standardized score [SE], 0.35 [0.05] vs 0.18 [0.06]; *P* = .03). There was a significant increase in male residents’ competency scores between PGY2 and PGY3 (range of difference in mean adjusted standardized scores between PGY2 and PGY3, 0.208-0.391; *P* ≤ .002) that was not seen in female residents’ scores (range of difference in mean adjusted standardized scores between PGY2 and PGY3, −0.117 to 0.101; *P* ≥ .14). There was a significant increase in male residents’ scores between PGY2 and PGY3 cohorts in 6 competencies with female faculty and in 4 competencies with male faculty. There was no significant change in female residents’ competency scores between PGY2 to PGY3 cohorts with male or female faculty. Interaction between faculty-resident gender dyad and PGY was significant in the patient care competency (β estimate [SE] for female vs male dyad in PGY1 vs PGY3, 0.184 [0.158]; β estimate [SE] for female vs male dyad in PGY2 vs PGY3, 0.457 [0.181]; *P* = .04).

**Conclusions and Relevance:**

In this study, resident gender was associated with differences in faculty assessments of resident performance, and differences were linked to PGY. In contrast to male residents’ scores, female residents’ scores displayed a peak-and-plateau pattern whereby assessment scores peaked in PGY2. Notably, the peak-and-plateau pattern was seen in assessments by male and female faculty. Further study of factors that influence gender-based differences in assessment is needed.

## Key Points

**Question:**

How is gender associated with faculty assessment of internal medicine resident performance?

**Findings:**

In this multisite cross-sectional study, resident gender was associated with differences in faculty assessments of resident performance, and differences were linked to postgraduate year. With both male and female faculty evaluators, female residents’ scores displayed a peak-and-plateau pattern whereby assessment scores peaked in postgraduate year 2.

**Meaning:**

These findings suggest that gender of trainees and faculty is associated with resident assessment.

## Teaser

This cross-sectional study evaluates the association of gender with assessment of internal medicine residents.

## Introduction

Implicit gender bias refers to how culturally established gender roles and beliefs unconsciously affect our perceptions and actions^1^ and may influence the continuum of the medical profession, including students,^2,3,4^ trainees,^5,6,7,8,9^ and practicing physicians.^10,11,12^ Gender bias has been cited as a potential threat to the integrity of resident assessment.^13^

Competency-based medical education as implemented in the Next Accreditation System of the Accreditation Council for Graduate Medical Education (ACGME) relies on meaningful assessment to inform judgments about resident progress.^14^ Bias in assessment is of heightened concern in competency-based medical education, given implications for resident time in training and readiness to practice.

Evidence of gender bias in resident assessment using the Next Accreditation System competency-based framework is limited.^5,15,16^ A 2017 study of emergency medicine training programs found that faculty ascribed higher Milestone levels to male residents at the end of training compared with their female peers.^5^ However, a 2019 national study of Milestones reported to the ACGME found that emergency medicine programs’ clinical competency committees reported similar Milestone levels for male and female residents with small but significant differences noted in 4 subcompetencies.^16^

The need to assess for gender bias within competency-based resident assessment is critical. This study examines the influence of gender on faculty assessment of resident performance in internal medicine residency training.

## Methods

We conducted a retrospective, cross-sectional study of faculty assessments of residents in 6 internal medicine residency training programs: Emory University, Atlanta, Georgia; Massachusetts General Hospital, Boston; University of Alabama, Birmingham; University of California, San Francisco; University of Chicago, Chicago, Illinois; and University of Louisville, Louisville, Kentucky. The institutional review board at each institution reviewed and approved the study protocol and waived the requirement of informed consent for this retrospective analysis. This study followed the Strengthening the Reporting of Observational Studies in Epidemiology (STROBE) reporting guideline.

Data included faculty assessments of internal medicine resident performance during general medicine inpatient rotations from July 1, 2016, to June 30, 2017. Participants included categorical and primary care internal medicine residents. Inpatient general medicine ward teams include a postgraduate year 2 (PGY2) or PGY3 resident overseeing a team of PGY1 residents under the supervision of 1 to 2 attendings. Residents engage in multiple inpatient general medicine rotations per year. Residents spend 2 weeks to 1 month on these rotations, while attendings rotate in 1-week to 1-month blocks. Faculty evaluate each resident under their supervision. Faculty assessments are used to inform overall performance evaluations of resident progress as reported to the ACGME using the Next Accreditation System framework for competency-based assessment.

Assessment data included 20 of 22 internal medicine–specific reporting Milestones and 6 core competencies (patient care, medical knowledge, systems-based practice [SBP], practice-based learning and improvement [PBLI], professionalism, and interpersonal and communication skills [ICS]). See eTable 1 in the Supplement for data collected for Milestones and core competencies across sites.^17^

Each site used a unique assessment tool, which, in aggregate, included 130 quantitative questions, 45 of which used exact wording of the ACGME’s reporting Milestones and 85 of which used variations of the Milestones wording. Eight team members (R.K., N.N.U., J.K., A.V., E.D.S., S.S., V.T., K.A.J.) independently and blindly matched question stems to the most appropriate Milestone (96% agreement), with disagreement resolved through discussion.

Rating scales varied across programs. To address this, we converted rating scores to a standardized score. Within a site, we calculated the rating distribution for each Milestone, including mean, distribution, and SD, then used these data to calculate standardized scores for that milestone. We calculated standardized scores for each Milestone and each core competency at each site and used standardized scores in aggregate for analysis. Standardized scores are expressed as SDs from the mean.

We also collected resident and faculty demographic data as well as rotation setting and date. Resident demographics included gender, PGY, and baseline internal medicine In-Training Examination (ITE) percentile rank, defined as the percentile rank on the first ITE examination required by each program. Faculty demographics included gender, specialty, academic rank, and residency educational role.

We used male and female gender designations, and gender was determined by participants’ professional gender identity. Demographic data were obtained from residency management systems and search of institution websites. The program director or associate program director at each site not involved in the study reviewed and verified gender designations. Data were deidentified before analysis.

### Statistical Analysis

Data were analyzed from June 7 to November 6, 2019. For all variables, we computed summary statistics and calculated standardized scores for each Milestone and core competency at each site and used standardized scores in aggregate for analysis. We evaluated the association of standardized scores for Milestones and core competencies with resident gender, PGY, and faculty gender with a random-intercept mixed model adjusted for clustering of residents and faculty within programs. After testing for the individual main effects of the 3 variables above, we assessed for the interaction of resident gender, PGY, and faculty gender. We adjusted for resident ITE percentile rank, faculty rank (professor, associate professor, assistant professor/instructor/chief resident, or no rank/clinical associate), faculty specialty (general medicine, hospital medicine, or subspecialty), rotation setting (university, Veterans Administration, public, or community hospital), and rotation time of year (July-September, October-December, January-March, or April-June). Analyses were conducted in SAS, version 9.4 (SAS Institute, Inc). A 2-sided *P* < .05 was considered statistically significant.

## Results

Data included 3600 assessments for 703 residents (387 male [55.0%] and 316 female [45.0%]) by 605 faculty members (318 male [52.6%] and 287 female [47.4%]). Table 1 details demographic data. There was no difference in baseline ITE by gender (mean [SE] ITE for male vs female residents, 67.0 [1.3] vs 62.2 [1.5]; *P* = .15) or PGY cohort (mean [SE] ITE, 64.6 [1.6] for PGY1; 63.5 [1.7] for PGY2; 66.6 [1.8] for PGY3; *P* = .45). There was a gender-based difference in baseline internal medicine ITE in the PGY3 cohort (mean [SE] ITE, 71.1 [2.4] for male residents vs 61.1 [2.7] for female residents; *P* = .006).

**Table 1.** Demographic Data for Residents, Faculty, and Assessments

Characteristic	Data^a^
**Residents assessed (n = 703)**	
Gender	
Male	387 (55.0)
Female	316 (45.0)
Postgraduate year	
1	269 (38.3)
2	226 (32.1)
3	208 (29.6)
Baseline internal medicine ITE percentile rank, mean (SE)	64.8 (1.0)
Male resident ITE	67.0 (1.3)
Female resident ITE	62.2 (1.5)
**Faculty completing assessments (n = 605)**	
Gender	
Male	318 (52.6)
Female	287 (47.5)
Faculty rank	
Professor	111 (18.3)
Associate professor	115 (19.0)
Assistant professor or instructor	323 (53.4)
Chief resident	30 (5.0)
No rank or clinical associate	26 (4.3)
Faculty department	
General medicine	239 (39.5)
Hospital medicine	223 (36.9)
Subspecialty	143 (23.6)
Faculty educational role	
Program director	8 (1.3)
Associate program director	35 (5.8)
Chief resident	31 (5.1)
**Assessments (n = 3600)**	
No. of assessments per resident, mean (SD)	5.1 (3.4)
PGY1	7.9 (3.7)
PGY2	3.8 (1.6)
PGY3	3.0 (1.5)
No. of assessments per faculty, mean (SD)	6.0 (4.7)
Site	
Site 1	1065 (29.6)
Site 2	927 (25.8)
Site 3	678 (18.8)
Site 4	387 (10.8)
Site 5	306 (8.5)
Site 6	237 (6.6)
Hospital setting	
University hospital	2016 (56.0)
Public hospital	639 (17.8)
Veterans administration hospital	622 (17.3)
Community hospital	323 (9.0)
Time of year assessed	
July to September	925 (25.7)
October to December	879 (24.4)
January to March	976 (27.1)
April to June	820 (22.8)
Faculty-resident dyad	
Male resident-male faculty	1074 (29.8)
Male resident-female faculty	867 (24.1)
Female resident-male faculty	909 (25.3)
Female resident-female faculty	750 (20.8)

Abbreviations: ITE, in-training examination; PGY, postgraduate year.

^a^ Unless otherwise indicated, data are expressed as number (percentage) of participants.

### Influence of Resident Gender

Table 2 details adjusted core competency standardized scores by resident gender and PGY. eTable 2 in the Supplement includes adjusted standardized Milestones scores.

**Table 2.** Adjusted Standardized Scores for Core Competencies in Post-Graduate Year Cohorts by Resident Gender

Core competency	PGY1			PGY2			PGY3			Overall *P* value^c^
	Mean (SE) score^a^		*P* value^b^	Mean (SE) score^a^		*P* value^b^	Mean (SE) score^a^		*P* value^b^	
	Male	Female		Male	Female		Male	Female		
Patient care	−0.15 (0.03)	−0.11 (0.03)	.31	0.10 (0.04)	0.22 (0.05)	.04	0.47 (0.05)	0.32 (0.05)	.03	.01
Medical knowledge	−0.28 (0.03)	−0.29 (0.04)	.82	0.26 (0.05)	0.32 (0.05)	.35	0.47 (0.05)	0.24 (0.06)	<.01	.01
SBP	−0.28 (0.03)	−0.25 (0.04)	.48	−0.06 (0.05)	0.13 (0.05)	.003	0.30 (0.05)	0.12 (0.06)	.02	<.01
PBLI	−0.13 (0.03)	−0.17 (0.04)	.34	0.08 (0.05)	0.15 (0.06)	.30	0.39 (0.05)	0.16 (0.06)	<.01	.02
Professionalism	−0.14 (0.04)	−0.09 (0.04)	.16	−0.04 (0.06)	0.21 (0.06)	<.01	0.35 0.05)	0.18 (0.06)	.03	<.001
ICS	−0.17 (0.04)	−0.10 (0.04)	.12	0.06 (0.05)	0.32 (0.06)	<.001	0.31 (0.06)	0.20 (0.07)	.23	<.01

Abbreviation: ICS, interpersonal and communication skills; PBLI, practice-based learning and improvement; PGY, postgraduate year; SBP, systems-based practice.

^a^ Indicates adjusted standardized scores for internal medicine core competencies as determined by the Accreditation Council for Graduate Medical Education.^17^ Scores were obtained from a random-intercept mixed model adjusted for the clustering of residents within faculty within programs and baseline internal medicine In-Training Examination percentile rank, time of year evaluated (July to September, October to December, January to March, or April to May), rotation setting (university, Veterans Administration, community, or public hospital), faculty rank (assistant professor/instructor/chief resident, associate professor, professor, or no rank/clinical associate), and faculty specialty (general medicine, hospital medicine, or subspecialty).

^b^ Indicates the significance of the difference in mean adjusted standard scores between male and female residents per PGY.

^c^ Indicates the significance of the association of adjusted standard scores with resident gender and PGY.

Resident gender was significantly associated with assessment scores. There was no substantial difference in competency scores between male and female PGY1 residents. Female PGY2 residents scored higher than their male peers in all competencies, reaching statistical significance in patient care (mean [SE] adjusted standardized score, 0.10 [0.04] vs 0.22 [0.05]; *P* = .04), SBP (mean [SE] adjusted standardized score, −0.06 [0.05] vs 0.13 [0.05]; *P* = .003), professionalism (mean [SE] adjusted standardized score, −0.04 [0.06] vs 0.21 [0.06]; *P* = .001), and ICS (mean [SE] adjusted standardized score, 0.06 [0.05] vs 0.32 [0.06]; *P* < .001). However, scores of PGY3 male residents were significantly higher than those of their female peers in patient care (mean [SE] adjusted standardized score, 0.47 [0.05] vs 0.32 [0.05]; *P* = .03), medical knowledge (mean [SE] adjusted standardized score, 0.47 [0.05] vs 0.24 [0.06]; *P* = .003), SBP (mean [SE] adjusted standardized score, 0.30 [0.05] vs 0.12 [0.06]; *P* = .02), PBLI (mean [SE] adjusted standardized score, 0.39 [0.05] vs 0.16 [0.06]; *P* = .004), and professionalism (mean [SE] adjusted standardized score, 0.35 [0.05] vs 0.18 [0.06]; *P* = .03). This pattern in which female residents scored higher in PGY2 and male residents scored higher in PGY3 was noted in unadjusted scores (eTables 3 and 4 in the Supplement) and across sites (eTable 5 in the Supplement).

**Figure 1.**
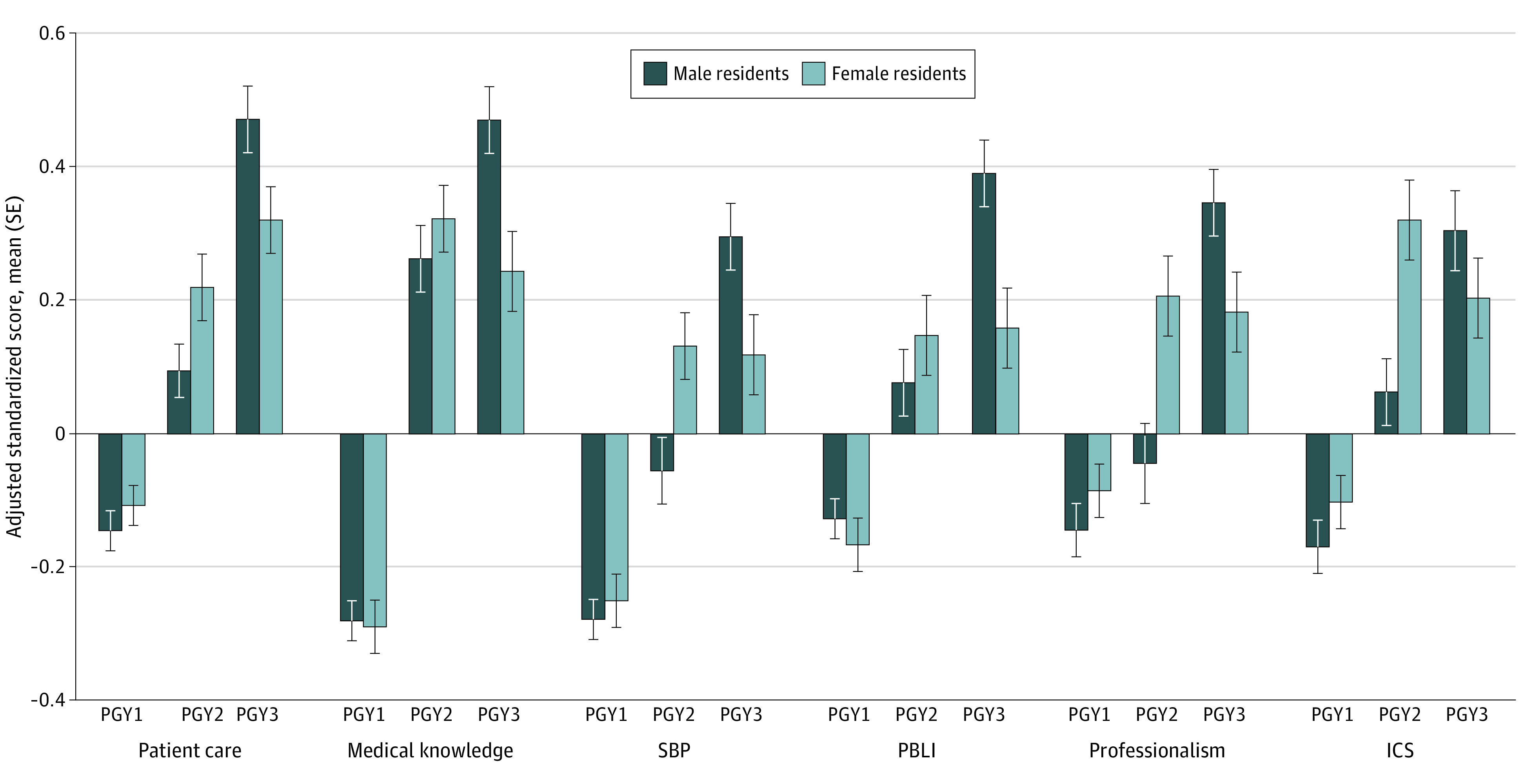
Adjusted Standardized Scores in the Core Competencies for Male and Female Internal Medicine Residents Data are stratified by postgraduate year (PGY). ICS indicates interpersonal and communication skills; PBLI, practice-based learning and improvement; and SBP, systems-based practice.

Figure 1 depicts standardized scores for PGY cohorts in the 6 competencies by resident gender. Male and female residents’ scores increased from PGY1 to PGY2 cohorts in all competencies. There was a significant positive difference in male residents’ adjusted standardized scores from PGY2 to PGY3 in patient care (0.377; *P* < .001), medical knowledge (0.208; *P* = .002), SBP (0.351; *P* < .001), PBLI (0.314; *P* < .001), professionalism (0.391; *P* < .001), and ICS (0.242; *P* = .002). Comparatively, the difference in adjusted standardized scores for female residents from PGY2 to PGY3 was nonsignificant for patient care (0.101; *P* = .14), medical knowledge (−0.079; *P* = .28), SBP (−0.013; *P* = .85), PBLI (0.011; *P* = .89), professionalism (−0.024; *P* = .77), and ICS (−0.117; *P* = .15). The interaction between resident gender and PGY was significant in all core competencies and 12 of 20 Milestones assessed in our study.

### Influence of Faculty Gender

**Figure 2.**
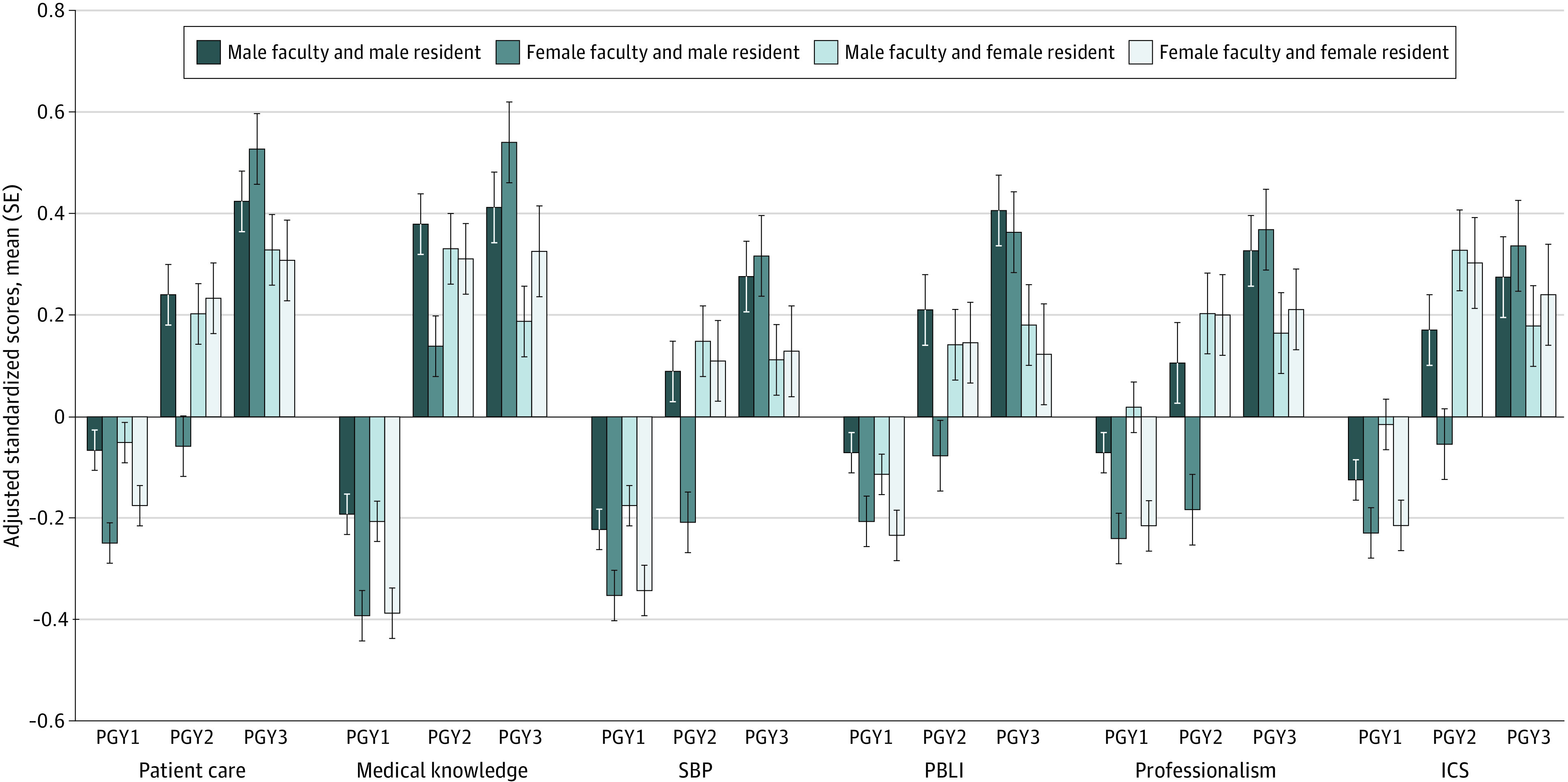
Adjusted Standardized Scores in the Core Competencies by Resident and Faculty Gender Data are stratified by postgraduate year (PGY). ICS indicates interpersonal and communication skills; PBLI, practice-based learning and improvement; and SBP, systems-based practice.

Figure 2 and Table 3 depict the adjusted standardized scores in core competencies for PGY cohorts by resident and faculty gender. With male faculty, there was no significant difference between male and female residents’ scores in PGY1 and PGY2 cohorts. Male faculty rated male PGY3 residents higher than female PGY3 residents in all competencies, reaching statistical significance in medical knowledge (mean [SE] adjusted standardized score, 0.42 [0.07] vs 0.19 [0.07]; *P* = .02) and PBLI (mean [SE] adjusted standardized score, 0.41 [0.07] vs 0.18 [0.08]; *P* = .03).

**Table 3.** Adjusted Standardized Scores in Core Competencies by Resident Gender, Faculty Gender, and Post-Graduate Year

Core competency	Male faculty									Female faculty									Overall *P* value^c^
	PGY1			PGY2			PGY3			PGY1			PGY2			PGY3			
	Mean (SE) score^a^		*P* value^b^	Mean (SE) score^a^		*P* value^b^	Mean (SE) score^a^		*P* value^b^	Mean (SE) score^a^		*P* value^b^	Mean (SE) score^a^		*P* value^b^	Mean (SE) score^a^		*P* value^b^	
	Male resident	Female resident		Male resident	Female resident		Male resident	Female resident		Male resident	Female resident		Male resident	Female resident		Male resident	Female resident		
Patient care	−0.07 (0.04)	−0.05 (0.04)	.77	0.24 (0.06)	0.20 (0.06)	.64	0.43 (0.06)	0.33 (0.07)	.30	−0.25 (0.04)	−0.18 (0.04)	.19	−0.06 (0.06)	0.24 (0.07)	<.001	0.53 (0.07)	0.31 (0.08)	.04	.04
Medical knowledge	−0.19 (0.04)	−0.21 (0.04)	.79	0.38 (0.06)	0.33 (0.07)	.57	0.42 (0.07)	0.19 (0.07)	.02	−0.39 (0.05)	−0.39 (0.05)	.93	0.14 (0.06)	0.31 (0.07)	.05	0.54 (0.08)	0.33 (0.09)	.06	.36
SBP	−0.22 (0.04)	−0.18 (0.04)	.38	0.09 (0.06)	0.15 (0.07)	.49	0.28 (0.07)	0.11 (0.07)	.09	−0.35 (0.05)	−0.34 (0.05)	.87	−0.21 (0.07)	0.11 (0.07)	<.001	0.39 (0.08)	0.13 (0.09)	.10	.12
PBLI	−0.07 (0.04)	−0.11 (0.04)	.43	0.21 (0.07)	0.14 (0.07)	.46	0.41 (0.07)	0.18 (0.08)	.03	−0.21 (0.05)	−0.24 (0.05)	.64	−0.08 (0.07)	0.15 (0.08)	.02	0.37 (0.08)	0.12 (0.10)	.05	.18
Professionalism	−0.07 (0.04)	0.02 (0.05)	.10	0.11 (0.08)	0.21 (0.08)	.37	0.33 (0.07)	0.17 (0.08)	.10	−0.24 (0.05)	−0.22 (0.05)	.68	−0.18 (0.07)	0.20 (0.09)	<.001	0.37 (0.08)	0.21 (0.09)	.17	.12
ICS	−0.13 (0.04)	−0.02 (0.05)	.05	0.17 (0.07)	0.33 (0.08)	.12	0.28 (0.08)	0.18 (0.08)	.37	−0.23 (0.05)	−0.22 (0.05)	.81	−0.05 (0.07)	0.31 (0.08)	<.001	0.34 (0.09)	0.24 (0.10)	.46	.21

Abbreviations: ICS, interpersonal and communication skill; PBLI, practice-based learning and improvement; PGY, postgraduate year; SBP, systems-based practice.

^a^ Indicates adjusted standardized scores for internal medicine core competencies as determined by the Accreditation Council for Graduate Medical Education.^17^ Scores were obtained from a random-intercept mixed model adjusted for the clustering of residents within faculty within programs and baseline internal medicine In-Training Examination percentile rank, time of year evaluated (July to September, October to December, January to March, or April to May), rotation setting (university, Veterans Administration, community, or public hospital), faculty rank (assistant professor/instructor/chief resident, associate professor, professor, or no rank/clinical associate), and faculty specialty (general medicine, hospital medicine, or subspecialty).

^b^ Compares mean adjusted standard scores between male and female residents per PGY within male and female faculty groups.

^c^ Compares the association of mean adjusted standard scores with resident gender, faculty gender, and PGY.

With female faculty, there was no significant difference in male and female PGY1 residents’ scores. Female faculty rated female PGY2 residents higher than male PGY2 residents in all competencies, reaching statistical significance in patient care (mean [SE] adjusted standardized score, −0.06 [0.06] vs 0.24 [0.07]; *P* < .001), SBP (mean [SE] adjusted standardized score, −0.21 [0.07] vs 0.11 [0.07]; *P* < .001), PBLI (mean [SE] adjusted standardized score, −0.08 [0.07] vs 0.15 [0.08]; *P* = .02), professionalism (mean [SE] adjusted standardized score, −0.18 [0.07] vs 0.20 [0.09]; *P* < .001), and ICS (mean [SE] adjusted standardized score, −0.05 [0.07] vs 0.31 [0.08]; *P* < .001). However, female faculty rated male PGY3 residents higher than female PGY3 residents in all competencies, reaching statistical significance in patient care (mean [SE] adjusted standardized score, 0.53 [0.07] vs 0.31 [0.08]; *P* = .04). Interaction between faculty-resident gender dyad and PGY was significant in the patient care competency (β estimate [SE] for female vs male dyad in PGY1 vs PGY3, 0.184 [0.158]; β estimate [SE] for female vs male dyad in PGY2 vs PGY3, 0.457 [0.181]; *P* = .04).

There was a significant increase in female residents’ standardized scores in all competencies from PGY1 to PGY2 with male (range in difference in mean adjusted standard score, 0.19-0.54; *P* ≤ .04) and female (range in difference in mean adjusted standard score, 0.38-0.70; *P* < .001) faculty. However, there was no significant difference in female residents’ scores across competencies between PGY2 and PGY3 with male (range in difference in mean adjusted standard score, −0.15 to 0.13; *P* ≥ .14) and female (range in difference in mean adjusted standard score, −0.063 to 0.08; *P* ≥ .46) faculty. In contrast, male residents’ scores significantly increased in all competencies between PGY2 and PGY3 with female faculty (range in difference in mean adjusted standard score, 0.39-0.59; *P* < .001) and in 4 of 6 competencies with male faculty (range in difference in mean adjusted standard score, 0.19-0.22; *P* ≤ .04).

In general, scores from male faculty were higher than those from female faculty regardless of resident gender. Overall, male residents’ scores were significantly higher from male faculty than from female faculty in patient care (mean [SE] adjusted standardized score, 0.20 [0.03] vs 0.04 [0.04]; *P* < .001), medical knowledge (mean [SE] adjusted standardized score, 0.21 [0.04] vs 0.05 [0.04]; *P* < .001), PBLI (mean [SE] adjusted standardized score, 0.17 [0.04] vs 0.02 [0.04]; *P* < .001), professionalism (mean [SE] adjusted standardized score, 0.12 [0.04] vs −0.035 [0.04]; *P* < .001), and ICS (mean [SE] adjusted standardized score, 0.11 [0.04] vs −0.001 [0.04]; *P* = .02). Female residents’ scores from male faculty were significantly higher than scores from female faculty in SBP (mean [SE] adjusted standardized score, 0.06 [0.04] vs −0.05 [0.04]; *P* = .03), professionalism (mean [SE] adjusted standardized score, 0.17 [0.04] vs 0.03 [0.04]; *P* = .008), and interpersonal and communication skills (mean [SE] adjusted standardized score, 0.19 [0.04] vs 0.08 [0.04]; *P* = .02).

## Discussion

To our knowledge, this is the first multisite quantitative study of the association of gender with assessment scores of internal medicine residents using a Milestone- and competency-based framework. Our findings indicate that (1) resident gender was a significant factor associated with assessment; (2) gender-based differences in assessment of internal medicine residents were associated with PGY; and (3) faculty gender was a notable factor associated with gender-based differences in assessment.

First, we found that resident gender was a significant factor associated with assessment. This is consistent with findings in assessment of emergency medicine residents.^5^ Many prior studies that did not show a gender-based difference in resident assessment^15,18,19,20,21^ were limited by low power, a low proportion of female participants, single-institution settings, or reliance on older assessment tools. A competency-based assessment framework did not appear to mitigate the influence of gender on faculty assessment of resident performance.

Second, we found that the gender-based differences in assessment of internal medicine residents were linked to PGY. Remarkably, the association of gender with assessment was not consistent across PGY cohorts. Male and female PGY1 residents scored similarly. In PGY2, when residents first assume the role of ward team leader, female residents earned higher marks than their male peers. However, this finding was reversed in PGY3, when male residents outscored female residents.

A peak-and-plateau pattern in female residents’ scores was noted whereby scores peaked in PGY2 and then did not improve beyond this level in PGY3 (Figure 1). Noted in all 6 competencies, the peak-and-plateau pattern of female residents’ scores contrasts with the positive trajectory of male residents’ scores. Studies that have indicated a link between time in training and gender-based differences in assessment have largely focused on gender-based differences at the end of training.^5,6,16^

This peak-and-plateau pattern may represent a glass ceiling in resident assessment. Traditionally reported in career advancement, the glass ceiling is a metaphor for invisible, unacknowledged barriers that become more pronounced at higher professional levels that impede the professional advancement of women and minorities.^22^ It is plausible that a phenomenon akin to the glass ceiling may manifest in residency, given its hierarchical nature.

In addition, we found that faculty gender was a notable factor in the gender-based differences in resident assessment. Gender-congruent resident faculty pairings seemed to benefit male residents more than female residents in terms of assessment scores. The peak-and-plateau pattern in female residents’ scores was noted with both male and female faculty evaluators. The interaction among resident gender, PGY, and faculty gender was significant in the patient care competency, which had the most assessment data in our study and is arguably the most summative competency. Interestingly, national study of Milestones reported by US emergency medicine programs also reported statistically significant differences in only patient care subcompetencies.^16^

Prior efforts to discern the association of faculty gender and gender pairings with resident assessment have yielded a limited picture.^5,6,7,18,19,21^ Of those studies that noted differences, findings suggest the male resident–male faculty dyad had higher scores than the female resident–male faculty dyad.^7,18^ We found gender-based differences in assessment with both male and female faculty. Evidence suggests that both women and men may display gender bias,^23,24^ and women’s own experiences with bias may influence this.^25^

Consideration must be given to potential sources of gender-based differences in assessment noted in our work. This includes the assessment framework, faculty evaluators, and resident learners. Gender-based differences in assessment have been reported using a variety of frameworks, including the Milestone- and competency-based assessment framework noted herein.^13^

Differing faculty expectations of residents may play a role in our findings. In our context, there is no explicit difference in the role of a PGY2 and PGY3 ward team leader in terms of responsibilities and duties. However, faculty may have different implicit expectations for PGY2 and PGY3 resident team leaders, which may enable implicit gender bias in assessment.

Gender bias may arise when gender-based normative behaviors and expectations misalign with professional roles and behaviors.^26^ It may emerge in specific contexts, such as a team leader role in which residents direct others in managing patient care. Research indicates that women successful in traditionally male fields may face a “likability penalty” that may impede career trajectory, which may explain the peak-and-plateau pattern we noted.^23,24,26^ Female residents are more often assessed using communal descriptors and less often in agentic terms.^8,9,27^ Female residents may be rewarded for adopting a communal leadership style in PGY2 and face a likability penalty for transitioning to a more assertive, independent leadership style in PGY3. A study of feedback provided to female emergency medicine PGY3 residents reported a faculty focus on autonomy, assertiveness, and receptiveness to oversight, which may suggest implicit faculty expectations around these issues for female residents.^6^

Not previously reported, we found that female faculty rated male PGY2 residents lower than female residents, but this reversed in PGY3 residents. Score patterns for male residents may reflect mismatch between confidence and competency or traits ascribed to the traditional male gender role. Evidence suggests male medical students and residents may overestimate confidence.^28,29,30^ Overestimation of confidence relative to competence may be seen as more detrimental in PGY2 than PGY3. Alternately, the traditional male gender role reinforces stoicism, independence, and less inclination to seek help, traits which may be seen as beneficial in PGY3 but not PGY2.^31^

Gender-based differences in assessment may reflect differences in resident performance. Given this study’s retrospective, cross-sectional design, it is possible that findings might reflect a difference between PGY cohorts. However, we noted this pattern across multiple sites in our study, suggesting that systematic differences between resident cohorts is less likely the root cause of the gender-based differences noted.

We incorporated baseline ITE percentile rank as an objective measure of baseline medical knowledge. Although we observed no significant overall gender-based difference in baseline ITE, we did note a difference in baseline ITE in PGY3 residents. This alone is likely insufficient to explain gender-based differences in assessments of resident performance. While associated with board certification pass rates, evidence supporting the ITE to estimate clinical performance is limited.^32,33^ Examining national trends in ITE by gender warrants further study.

Given variable expression in training, it seems unlikely that gender-based differences in scores are solely explained by deficiencies in clinical skill. Although evidence suggests that female residents experience strain when their professional role requires them to act counter to gender-based normative behaviors, it is unclear whether this affects performance.^34^ Finally, discordant, nonspecific feedback received by female residents may affect growth trajectory.^6^

We must consider the potential implications of these findings in graduate medical education. Because faculty assessment informs program determinations of resident progress, gender-based differences in assessment may have implications for resident time in training and readiness to practice.^5^ Faculty assessment data may influence professional opportunities accessible to residents.^13^ Finally, gender-based differences in assessment imply a difference in the training experience of male and female residents. Any evidence of disparities in training warrant attention and remedy.

### Limitations

Study limitations include the retrospective, cross-sectional approach. Differences between resident groups and variability in evaluation numbers between sites may influence findings. Although reproducibility across sites strengthen our findings, longitudinal study is warranted. Variability in assessment tools across sites was a limitation, although we used a rigorous approach to enable comparison. We used binary gender designations determined by participants’ professional gender identity, which does not adequately capture those identifying as gender nonbinary. Other factors, such as race and time spent observing resident performance may influence assessment; study of these factors is ongoing. Finally, our study included academic training programs, which may limit generalizability.

## Conclusions

Our study provides novel evidence of and insights into gender bias in assessment in graduate medical education. Further study of the factors that underlie gender-based differences in assessment is warranted to inform evidence-based interventions to address gender-based differences in assessment.

## Notes

Supplement. **eTable 1.** Core Competencies and Milestones Assessed per Site **eTable 2.** Adjusted Standardized Scores in Internal Medicine Milestones by Resident Gender and PGY **eTable 3.** Unadjusted Standardized Scores in Internal Medicine Core Competencies by Resident Gender and PGY **eTable 4.** Unadjusted Standardized Scores in Internal Medicine Milestones by Resident Gender and PGY **eTable 5.** Adjusted Standardized Scores for Core Competencies by Resident Gender and PGY at Each Site

## References

[1] Risberg G, Johansson EE, Hamberg K A theoretical model for analysing gender bias in medicine. Int J Equity Health. 2009;8(1):28. doi:10.1186/1475-9276-8-28 19646289 PMC2731093

[2] Axelson RD, Solow CM, Ferguson KJ, Cohen MB Assessing implicit gender bias in Medical Student Performance Evaluations. Eval Health Prof. 2010;33(3):365-385. doi:10.1177/0163278710375097 20801977

[3] Ross DA, Boatright D, Nunez-Smith M, Jordan A, Chekroud A, Moore EZ Differences in words used to describe racial and gender groups in Medical Student Performance Evaluations. PLoS One. 2017;12(8):e0181659. doi:10.1371/journal.pone.0181659 28792940 PMC5549898

[4] Rojek AE, Khanna R, Yim JWL, Differences in narrative language in evaluations of medical students by gender and under-represented minority status. J Gen Intern Med. 2019;34(5):684-691. doi:10.1007/s11606-019-04889-9 30993609 PMC6502922

[5] Dayal A, O’Connor DM, Qadri U, Arora VM Comparison of male vs female resident milestone evaluations by faculty during emergency medicine residency training. JAMA Intern Med. 2017;177(5):651-657. doi:10.1001/jamainternmed.2016.9616 28264090 PMC5818781

[6] Mueller AS, Jenkins TM, Osborne M, Dayal A, O’Connor DM, Arora VM Gender differences in attending physicians’ feedback to residents: a qualitative analysis. J Grad Med Educ. 2017;9(5):577-585. doi:10.4300/JGME-D-17-00126.1 29075375 PMC5646913

[7] Rand VE, Hudes ES, Browner WS, Wachter RM, Avins AL Effect of evaluator and resident gender on the American Board of Internal Medicine evaluation scores. J Gen Intern Med. 1998;13(10):670-674. doi:10.1046/j.1525-1497.1998.00202.x 9798813 PMC1500895

[8] Galvin SL, Parlier AB, Martino E, Scott KR, Buys E Gender bias in nurse evaluations of residents in obstetrics and gynecology. Obstet Gynecol. 2015;126(suppl 4):7S-12S. doi:10.1097/AOG.0000000000001044 26375558

[9] Gerull KM, Loe M, Seiler K, McAllister J, Salles A Assessing gender bias in qualitative evaluations of surgical residents. Am J Surg. 2019;217(2):306-313. doi:10.1016/j.amjsurg.2018.09.029 30343879 PMC8687875

[10] Jena AB, Olenski AR, Blumenthal DM Sex differences in physician salary in US public medical schools. JAMA Intern Med. 2016;176(9):1294-1304. doi:10.1001/jamainternmed.2016.3284 27400435 PMC5558151

[11] Jena AB, Khullar D, Ho O, Olenski AR, Blumenthal DM Sex differences in academic rank in US medical schools in 2014. JAMA. 2015;314(11):1149-1158. doi:10.1001/jama.2015.10680 26372584 PMC4665995

[12] Salles A, Awad M, Goldin L, Estimating implicit and explicit gender bias among health care professionals and surgeons. JAMA Netw Open. 2019;2(7):e196545. doi:10.1001/jamanetworkopen.2019.6545 31276177 PMC12064094

[13] Klein R, Julian KA, Snyder ED, ; From the Gender Equity in Medicine (GEM) workgroup Gender bias in resident assessment in graduate medical education: review of the literature. J Gen Intern Med. 2019;34(5):712-719. doi:10.1007/s11606-019-04884-0 30993611 PMC6502889

[14] Holmboe ES, Sherbino J, Long DM, Swing SR, Frank JR The role of assessment in competency-based medical education. Med Teach. 2010;32(8):676-682. doi:10.3109/0142159X.2010.500704 20662580

[15] Acuña J, Situ-LaCasse EH, Patanwala AE, Identification of gender differences in ultrasound milestone assessments during emergency medicine residency training: a pilot study. Adv Med Educ Pract. 2019;10:141-145. doi:10.2147/AMEP.S196140 31114417 PMC6474641

[16] Santen SA, Yamazaki K, Holmboe ES, Yarris LM, Hamstra SJ Comparison of male and female resident milestone assessments during emergency medicine residency training. Acad Med. 2020;95(2):263-268. doi:10.1097/ACM.0000000000002988 31517688 PMC7004441

[17] Accreditation Council for Graduate Medical Education, American Board of Internal Medicine. The Internal Medicine Milestone Project Published July 2015. Accessed July 31, 2019. https://www.acgme.org/Portals/0/PDFs/Milestones/InternalMedicineMilestones.pdf

[18] Brienza RS, Huot S, Holmboe ES Influence of gender on the evaluation of internal medicine residents. J Womens Health (Larchmt). 2004;13(1):77-83. doi:10.1089/154099904322836483 15006280

[19] Thackeray EW, Halvorsen AJ, Ficalora RD, Engstler GJ, McDonald FS, Oxentenko AS The effects of gender and age on evaluation of trainees and faculty in gastroenterology. Am J Gastroenterol. 2012;107(11):1610-1614. doi:10.1038/ajg.2012.139 23160284

[20] Holmboe ES, Huot SJ, Brienza RS, Hawkins RE The association of faculty and residents’ gender on faculty evaluations of internal medicine residents in 16 residencies. Acad Med. 2009;84(3):381-384. doi:10.1097/ACM.0b013e3181971c6d 19240452

[21] Sulistio MS, Khera A, Squiers K, Effects of gender in resident evaluations and certifying examination pass rates. BMC Med Educ. 2019;19(1):10. doi:10.1186/s12909-018-1440-7 30616651 PMC6322320

[22] Cotter DA, Hermsen JM, Ovadia S, Vanneman R The glass ceiling effect. Soc Forces. 2001;80(2):655-681. doi:10.1353/sof.2001.0091

[23] Heilman ME, Wallen AS, Fuchs D, Tamkins MM Penalties for success: reactions to women who succeed at male gender-typed tasks. J Appl Psychol. 2004;89(3):416-427. doi:10.1037/0021-9010.89.3.416 15161402

[24] Heilman ME Gender stereotypes and workplace bias. Res Organ Behav. 2012;32:113-135. doi:10.1016/j.riob.2012.11.003

[25] Ellemers N, van den Heuvel H, de Gilder D, Maass A, Bonvini A The underrepresentation of women in science: differential commitment or the queen bee syndrome? Br J Soc Psychol. 2004;43(pt 3):315-338. doi:10.1348/0144666042037999 15479533

[26] Eagly AH, Karau SJ Role congruity theory of prejudice toward female leaders. Psychol Rev. 2002;109(3):573-598. doi:10.1037/0033-295X.109.3.573 12088246

[27] Loeppky C, Babenko O, Ross S Examining gender bias in the feedback shared with family medicine residents. Educ Prim Care. 2017;28(6):319-324. doi:10.1080/14739879.2017.1362665 28812957

[28] Blanch DC, Hall JA, Roter DL, Frankel RM Medical student gender and issues of confidence. Patient Educ Couns. 2008;72(3):374-381. doi:10.1016/j.pec.2008.05.021 18656322

[29] Nomura K, Yano E, Fukui T Gender differences in clinical confidence: a nationwide survey of resident physicians in Japan. Acad Med. 2010;85(4):647-653. doi:10.1097/ACM.0b013e3181d2a796 20354381

[30] Lind DS, Rekkas S, Bui V, Lam T, Beierle E, Copeland EM III Competency-based student self-assessment on a surgery rotation. J Surg Res. 2002;105(1):31-34. doi:10.1006/jsre.2002.6442 12069498

[31] Schwab JR, Addis ME, Reigeluth CS, Berger JL Silence and (in)visibility in men’s accounts of coping with stressful life events. Gend Soc. 2015;30(2):289-311. doi:10.1177/0891243215602923

[32] Schwartz RW, Donnelly MB, Sloan DA, Johnson SB, Strodel WE The relationship between faculty ward evaluations, OSCE, and ABSITE as measures of surgical intern performance. Am J Surg. 1995;169(4):414-417. doi:10.1016/S0002-9610(99)80187-1 7694980

[33] Babbott SF, Beasley BW, Hinchey KT, Blotzer JW The predictive validity of the internal medicine in-training examination [published correction appears in *Am J Med* 2007;120(10):911]. Am J Med. 2007;120(8):735-740. doi:10.1016/j.amjmed.2007.05.003 17679136

[34] Kolehmainen C, Brennan M, Filut A, Isaac C, Carnes M Afraid of being “witchy with a “b”: a qualitative study of how gender influences residents’ experiences leading cardiopulmonary resuscitation. Acad Med. 2014;89(9):1276-1281. doi:10.1097/ACM.0000000000000372 24979289 PMC4146658

