# Supplementary material for: Association of Gender With Learner Assessment in Graduate Medical Education
Source: JAMA Netw Open. 2020 Jul 16;3(7):e2010888. doi: 10.1001/jamanetworkopen.2020.10888 (PMC7366188; doi:10.1001/jamanetworkopen.2020.10888)
Supplement: Supplement. — eTable 1. Core Competencies and Milestones Assessed per Site eTable 2. Adjusted Standardized Scores in Internal Medicine Milestones by Resident Gender and PGY eTable 3. Unadjusted Standardized Scores in Internal Medicine Core Competencies by Resident Gender and PGY eTable 4. Unadjusted Standardized Scores in Internal Medicine Milestones by Resident Gender and PGY eTable 5. Adjusted Standardized Scores for Core Competencies by Resident Gender and PGY at Each Site [file jamanetwopen-3-e2010888-s001.pdf]

Supplementary Online Content

Klein R, Ufere NN, Rao SR, et al. Association of gender with learner assessment in graduate medical education. *JAMA Netw Open*. 2020;3(7):e2010888. doi:10.1001/jamanetworkopen.2020.10888

eTable 1. Core Competencies and Milestones Assessed per Site

eTable 2. Adjusted Standardized Scores in Internal Medicine Milestones by Resident Gender and PGY

eTable 3. Unadjusted Standardized Scores in Internal Medicine Core Competencies by Resident Gender and PGY

eTable 4. Unadjusted Standardized Scores in Internal Medicine Milestones by Resident Gender and PGY

eTable 5. Adjusted Standardized Scores for Core Competencies by Resident Gender and PGY at Each Site

This supplementary material has been provided by the authors to give readers additional information about their work.

©2020 Klein R et al. *JAMA Network Open*.

eTable 1. Core Competencies and Milestones Assessed per Site

			Site 1	Site 2	Site 3	Site 4	Site 5	Site 6	Total
Assessments per site, N			1065	927	678	387	306	237	3600
**Core Competency & Milestone***		**Description**							
Patient Care			1063	927	678	387	305	237	3597
	PC1	Gathers and synthesizes essential and correct information to define each patient's clinical problem (s).	831	927	309	387	303	237	2994
	PC2	Develops and achieves comprehensive management plan for each patient.	960	927	292	385	304	236	3104
	PC3	Manages patients with progressive responsibility and independence.	656	366	306	173	305	236	2042
	PC4	Skill in performing procedures.	NA	NA	NA	NA	NA	NA	NA
	PC5	Requests and supplies consultative care.	NA	366	NA	175	301	237	1079
Medical Knowledge			1059	927	678	387	305	237	3593
	MK1	Clinical knowledge	960	927	678	387	305	237	3494
	MK2	Knowledge of diagnostic testing and procedures.	99	927	198	383	305	236	2148
Systems-Based Practice			1052	927	674	386	305	237	3581
	SBP1	Works effectively within an interprofessional team (e.g. peers, consultants, nursing, ancillary professionals and other support personnel).	868	927	369	384	305	236	3089
	SBP2	Recognizes system error and advocates for system improvement.	88	NA	136	337	NA	237	798
	SBP3	Finds forces that impact the cost of health care, and advocates for, and practices cost‐effective care.	294	NA	249	373	NA	236	1152
	SBP4	Transitions patients effectively within and across health delivery systems.	397	927	479	379	304	232	2718
Practice-Based Learning and Improvement			1045	927	674	387	NA	237	3270
	PBLI1	Monitors practice with a goal for improvement.	394	NA	75	NA	NA	236	705
	PBLI2	Learns and improves via performance audit.	NA	NA	NA	NA	NA	NA	NA

©2020 Klein R et al. *JAMA Network Open*.

	PBLI3	Learns and improves via feedback.	952	927	464	380	NA	235	2958
	PBLI4	Learns and improves at the point of care.	303	927	135	385	NA	235	1985
Professionalism			760	927	678	387	303	237	3292
	PROF1	Has professional and respectful interactions with patients, caregivers, and members of the interprofessional team (e.g. peers, consultants, nursing, ancillary professionals, and support personnel).	659	927	251	387	303	234	2761
	PROF2	Accepts responsibility and follows through on tasks.	653	NA	403	174	NA	236	1466
	PROF3	Responds to each patient's unique characteristics and needs.	98	NA	256	206	NA	NA	560
	PROF4	Exhibits integrity and ethical behavior in professional conduct.	562	561	355	NA	NA	237	1715
Interpersonal & Communication Skills			1060	927	543	386	303	NA	3219
	ICS1	Communicates effectively with patients and caregivers.	961	927	466	NA	303	NA	2657
	ICS2	Communicates effectively in interprofessional teams (e.g. peers, consultants, nursing, ancillary professionals, and other support personnel).	401	NA	198	NA	NA	NA	599
	ICS3	Appropriate use and completion of health records.	96	927	273	386	NA	NA	1682

Abbreviations: IM=Internal Medicine;; ICS=Interpersonal Communication & Skills; MK=Medical Knowledge; NA=Not Assessed PBLI=Practice-Based Learning & Improvement; PC=Patient Care; PROF=Professionalism; SBP=Systems-Based Practice;
*Core Competencies and Milestones for Internal Medicine Residency Training as determined by the Accreditation Council for Graduate Medical Education[17]

©2020 Klein R et al. *JAMA Network Open*.

eTable 2. Adjusted Standardized Scores in Internal Medicine Milestones by Resident Gender and PGY

IM Milestones*	Post-graduate year 1			Post-graduate year 2			Post-graduate year 3			Overall
	Male	Female	p[a]	Male	Female	p[a]	Male	Female	p[a]	p[b]
	Mean (SE)[±]			Mean (SE)[±]			Mean (SE)[±]			
**Patient Care**										
PC1	-0.067 (0.04)	-0.003 (0.04)	0.13	-0.317 (0.06)	-0.364 (0.08)	0.58	0.229 (0.06)	0.040 (0.07)	0.03	0.02
PC2	-0.254 (0.04)	-0.225 (0.04)	0.52	0.229 (0.05)	0.331 (0.06)	0.13	0.500 (0.06)	0.317 (0.07)	0.03	0.03
PC3	-0.282 (0.06)	-0.268 (0.06)	0.82	0.126 (0.06)	0.255 (0.08)	0.11	0.629 (0.06)	0.431 (0.06)	0.01	0.01
PC4	NA	NA	NA	NA	NA	NA	NA	NA	NA	NA
PC5	-0.046 (0.11)	0.012 (0.13)	0.67	-0.111 (0.08)	0.103 (0.10)	0.05	0.287 (0.09)	0.167 (0.09)	0.19	0.06
**Medical Knowledge**										
MK1	-0.241 (0.04)	-0.246 (0.04)	0.9	0.273 (0.05)	0.387 (0.05)	0.08	0.582 (0.06)	0.351 (0.07)	<0.01	<0.01
MK2	-0.375 (0.05)	-0.346 (0.05)	0.61	0.081 (0.07)	0.062 (0.07)	0.84	0.375 (0.06)	0.138 (0.07)	<0.01	0.03
**Systems- Based Practice**										
SBP1	-0.208 (0.04)	-0.201 (0.05)	0.88	0.032 (0.05)	0.217 (0.06)	<0.01	0.384 (0.07)	0.227 (0.07)	0.08	<0.01
SBP2	-0.103 (0.09)	0.206 (0.12)	0.02	-0.350 (0.11)	-0.107 (0.12)	0.11	0.232 (0.09)	-0.128 (0.10)	<0.01	<0.01
SBP3	-0.239 (0.06)	-0.118 (0.07)	0.19	-0.021 (0.08)	0.035 (0.09)	0.57	0.328 (0.08)	-0.017 (0.10)	<0.01	0.01
SBP4	-0.248 (0.04)	-0.171 (0.04)	0.14	0.008 (0.05)	0.223 (0.06)	<0.01	0.362 (0.06)	0.279 (0.07)	0.35	0.03
**Practice- Based Learning & Improvement**										
PBLI1	-0.392 (0.12)	-0.407 (0.15)	0.92	-0.161 (0.11)	-0.115 (0.10)	0.66	0.436 (0.11)	0.106 (0.15)	0.05	0.16
PBLI2	NA	NA	NA	NA	NA	NA	NA	NA	NA	NA
PBLI3	-0.139 (0.04)	-0.133 (0.04)	0.89	0.178 (0.06)	0.359 (0.07)	0.03	0.387 (0.07)	0.074 (0.08)	<0.01	<0.01
PBLI4	-0.147 (0.05)	-0.280 (0.06)	0.04	0.057 (0.06)	0.053 (0.07)	0.96	0.333 (0.07)	0.153 (0.08)	0.07	0.32
**Professionalism**										
PROF1	-0.097 (0.04)	-0.081 (0.04)	0.74	-0.042 (0.07)	0.248 (0.08)	<0.01	0.423 (0.06)	0.251 (0.07)	0.05	<0.01
PROF2	-0.035 (0.06)	0.031 (0.06)	0.25	0.289 (0.14)	0.246 (0.23)	0.87	0.486 (0.09)	0.170 (0.11)	0.02	0.04
PROF3	-0.411 (0.10)	-0.144 (0.12)	0.04	-0.355 (0.18)	-0.404 (0.18)	0.81	0.323 (0.18)	0.103 (0.19)	0.23	0.10
PROF4	-0.223 (0.05)	-0.183 (0.05)	0.44	0.166 (0.10)	0.351 (0.10)	0.17	0.657 (0.13)	0.761 (0.25)	0.7	0.59

©2020 Klein R et al. *JAMA Network Open*.

IM Milestones*	Post-graduate year 1			Post-graduate year 2			Post-graduate year 3			Overall
	Male	Female	p[a]	Male	Female	p[a]	Male	Female	p[a]	p[b]
	Mean (SE)±			Mean (SE)±			Mean (SE)±			
**Interpersonal & Communication Skills**										
ICS1	-0.178 (0.04)	-0.110 (0.04)	0.17	0.094 (0.06)	0.383 (0.07)	<0.001	0.147 (0.10)	0.340 (0.09)	0.27	0.06
ICS2	-0.693 (0.15)	-0.547 (0.15)	0.36	-0.074 (0.12)	0.027 (0.12)	0.43	0.753 (0.22)	0.529 (0.23)	0.28	0.33
ICS3	-0.140 (0.05)	-0.062 (0.05)	0.19	-0.005 (0.09)	0.131 (0.14)	0.38	0.187 (0.08)	0.040 (0.09)	0.19	0.18

Abbreviations: IM= Internal Medicine; ICS=Interpersonal Communication & Skill; MK=Medical Knowledge; NA=not assessed; p=p-value; PBLI=Problem-Based Learning & Improvement; PROF=Professionalism; SBP=Systems-Based Practice; SE=standard error; PC=Patient Care,

*Adjusted standardized scores for IM reporting Milestones as determined by the Accreditation Council for Graduate Medical Education[17]

±Mean and Standard Errors obtained from a random-intercept mixed model adjusted for the clustering of residents within faculty within programs and baseline IM In-Training Examination Percentile Rank, time of year evaluated (Jul-Sep, Oct-Dec, Jan-Mar, Apr-May), rotation setting (University, Veterans Administration, Community or Public hospital), faculty rank (Assistant Professor/Instructor/Chief Resident, Associate Professor, Professor, No Rank/Clinical Associate), and faculty specialty (General Medicine, Hospital Medicine, Subspecialty)

[a] p-value represents significance of the differences in mean adjusted standard scores between male and female residents per post-graduate year

[b] p-value represents significance of the association of mean standard score with resident gender and post-graduate year

©2020 Klein R et al. *JAMA Network Open*.

eTable 3. Unadjusted Standardized Scores in Internal Medicine Core Competencies by Resident Gender and PGY

IM Core Competencies*	Post-graduate year 1		Post-graduate year 2		Post-graduate year 3		
	Male	Female	Male	Female	Male	Female	P[a]
	Mean (SE)		Mean (SE)		Mean (SE)		
**Patient Care**	-0.141 (0.03)	-0.099 (0.03)	0.121 (0.04)	0.152 (0.04)	0.455 (0.05)	0.243 (0.05)	0.01
**Medical Knowledge**	-0.245 (0.03)	-0.251 (0.03)	0.333 (0.04)	0.300 (0.05)	0.481 (0.05)	0.177 (0.06)	<0.01
**Systems- Based Practice**	-0.208 (0.03)	-0.176 (0.03)	0.051 (0.04)	0.167 (0.05)	0.320 (0.05)	0.121 (0.06)	0.01
**Practice- Based Learning & Improvement**	-0.122 (0.03)	-0.148 (0.03)	0.121 (0.04)	0.132 (0.05)	0.383 (0.05)	0.107 (0.06)	0.01
**Professionalism**	-0.112 (0.03)	-0.051 (0.03)	0.025 (0.05)	0.188 (0.05)	0.355 (0.05)	0.162 (0.06)	<0.01
**Interpersonal & Communication Skills**	-0.156 (0.03)	-0.089 (0.03)	0.107 (0.05)	0.270 (0.05)	0.315 (0.06)	0.147 (0.06)	0.01

Abbreviations: IM= Internal Medicine; ICS=Interpersonal Communication & Skill; MK=Medical Knowledge; NA=not assessed; p=p-value; PBLI=Problem-Based Learning & Improvement; PROF=Professionalism; SBP=Systems-Based Practice; SE=standard error; PC=Patient Care,
*Standardized scores for IM reporting Milestones and Core Competencies as determined by the Accreditation Council for Graduate Medical Education[17]
[a] p-value represents significance of the association of mean standard score with resident gender and post-graduate year

©2020 Klein R et al. *JAMA Network Open*.

eTable 4. Unadjusted Standardized Scores in Internal Medicine Milestones by Resident Gender and PGY

IM Milestones*	Post-graduate year 1		Post-graduate year 2		Post-graduate year 3		
	Male	Female	Male	Female	Male	Female	P[a]
	Mean (SE)		Mean (SE)		Mean (SE)		
**Patient Care**							
PC1	-0.002 (0.03)	0.073 (0.03)	-0.176 (0.06)	-0.311 (0.07)	0.276 (0.06)	0.023 (0.06)	<0.01
PC2	-0.259 (0.03)	-0.229 (0.03)	0.268 (0.05)	0.310 (0.05)	0.451 (0.06)	0.197 (0.06)	0.01
PC3	-0.378 (0.04)	-0.376 (0.05)	0.052 (0.05)	0.087 (0.06)	0.571 (0.05)	0.339 (0.06)	0.03
PC4	NA	NA	NA	NA	NA	NA	NA
PC5	-0.175 (0.08)	0.102 (0.11)	-0.108 (0.06)	0.041 (0.08)	0.190 (0.06)	0.023 (0.07)	0.07
**Medical Knowledge**							
MK1	-0.242 (0.03)	-0.242 (0.03)	0.298 (0.04)	0.309 (0.05)	0.513 (0.06)	0.202 (0.06)	<0.01
MK2	-0.259 (0.04)	-0.229 (0.04)	0.264 (0.06)	0.122 (0.07)	0.465 (0.06)	0.160 (0.06)	0.01
**Systems- Based Practice**							
SBP1	-0.171 (0.03)	-0.166 (0.04)	0.105 (0.05)	0.223 (0.05)	0.369 (0.06)	0.202 (0.07)	0.04
SBP2	-0.092 (0.07)	0.261 (0.10)	-0.197 (0.10)	-0.050 (0.10)	0.205 (0.07)	-0.197 (0.09)	<0.01
SBP3	-0.259 (0.06)	-0.145 (0.07)	0.123 (0.07)	0.107 (0.07)	0.359 (0.08)	-0.040 (0.10)	0.01
SBP4	-0.229 (0.03)	-0.139 (0.04)	0.085 (0.05)	0.239 (0.05)	0.347 (0.06)	0.226 (0.06)	0.05
**Practice- Based Learning & Improvement**							
PBLI1	-0.349 (0.09)	-0.367 (0.12)	-0.063 (0.07)	-0.076 (0.07)	0.594 (0.09)	0.326 (0.12)	0.37
PBLI2	NA	NA	NA	NA	NA	NA	NA
PBLI3	-0.131 (0.03)	-0.113 (0.03)	0.228 (0.05)	0.388 (0.07)	0.344 (0.06)	-0.014 (0.08)	<0.01
PBLI4	-0.128 (0.04)	-0.230 (0.05)	0.134 (0.05)	0.054 (0.06)	0.329 (0.06)	0.087 (0.07)	0.39
**Professionalism**							
PROF1	-0.141 (0.03)	-0.112 (0.04)	0.017 (0.06)	0.230 (0.07)	0.394 (0.06)	0.196 (0.06)	0.01
PROF2	-0.086 (0.04)	-0.001 (0.04)	0.234 (0.14)	0.083 (0.23)	0.312 (0.08)	-0.057 (0.11)	0.01
PROF3	-0.159 (0.07)	0.077 (0.09)	-0.005 (0.14)	-0.016 (0.14)	0.253 (0.12)	0.028 (0.13)	0.11

©2020 Klein R et al. *JAMA Network Open*.

IM Milestones*	Post-graduate year 1		Post-graduate year 2		Post-graduate year 3		
	Male	Female	Male	Female	Male	Female	P[a]
	Mean (SE)		Mean (SE)		Mean (SE)		
PROF4	-0.097 (0.04)	-0.061 (0.04)	0.283 (0.09)	0.373 (0.10)	0.590 (0.12)	0.748 (0.25)	0.86
**Interpersonal & Communication Skills**							
ICS1	-0.144 (0.03)	-0.085 (0.03)	0.137 (0.05)	0.355 (0.06)	0.197 (0.09)	0.267 (0.09)	0.23
ICS2	-0.362 (0.10)	-0.296 (0.10)	-0.014 (0.08)	0.080 (0.08)	0.557 (0.13)	0.342 (0.14)	0.38
ICS3	-0.096 (0.04)	-0.022 (0.04)	0.131 (0.08)	0.201 (0.13)	0.240 (0.07)	-0.024 (0.08)	0.03

Abbreviations: IM= Internal Medicine; ICS=Interpersonal Communication & Skill; MK=Medical Knowledge; NA=not assessed; p=p-value; PBLI=Problem-Based Learning & Improvement; PROF=Professionalism; SBP=Systems-Based Practice; SE=standard error; PC=Patient Care,

* IM reporting Milestones as determined by the Accreditation Council for Graduate Medical Education[17]

[a] p-value represents significance of the association of mean standard score with resident gender and post-graduate year

©2020 Klein R et al. *JAMA Network Open*.

eTable 5. Adjusted Standardized Scores for Core Competencies by Resident Gender and PGY at Each Site

		Post-graduate year 1		Post-graduate year 2		Post-graduate year 3	
	**Core Competency***	**Male**	**Female**	**Male**	**Female**	**Male**	**Female**
		**Mean (SE)±**		**Mean (SE)±**		**Mean (SE)±**	
Site 1	Patient Care	-0.062 (0.04)	-0.034 (0.05)	0.244 (0.07)	0.137 (0.08)	0.721 (0.11)	0.576 (0.12)
	Medical Knowledge	-0.164 (0.05)	-0.201 (0.05)	0.538 (0.08)	0.520 (0.09)	0.160 (0.13)	-0.074 (0.14)
	Systems- Based Practice	-0.238 (0.05)	-0.219 (0.05)	0.309 (0.08)	0.296 (0.09)	0.230 (0.13)	-0.204 (0.14)
	Practice- Based Learning & Improvement	-0.158 (0.05)	-0.190 (0.05)	0.160 (0.08)	0.213 (0.09)	0.723 (0.13)	0.328 (0.14)
	Professionalism	-0.023 (0.05)	-0.025 (0.05)	0.536 (0.42)	0.369 (0.54)	0.596 (0.13)	0.233 (0.14)
	Interpersonal & Communication Skills	-0.192 (0.05)	-0.170 (0.05)	0.194 (0.08)	0.227 (0.09)	0.523 (0.13)	0.309 (0.14)
Site 2	Patient Care	-0.125 (0.05)	-0.050 (0.05)	-0.079 (0.07)	0.085 (0.11)	0.159 (0.1)	0.168 (0.09)
	Medical Knowledge	-0.254 (0.05)	-0.190 (0.05)	0.181 (0.07)	0.220 (0.11)	0.361 (0.1)	0.328 (0.1)
	Systems- Based Practice	-0.032 (0.05)	0.040 (0.05)	-0.184 (0.08)	0.017 (0.12)	0.099 (0.1)	0.187 (0.1)
	Practice- Based Learning & Improvement	-0.054 (0.05)	-0.123 (0.05)	0.097 (0.07)	0.097 (0.11)	0.214 (0.1)	0.164 (0.09)
	Professionalism	-0.033 (0.05)	0.031 (0.05)	-0.140 (0.08)	0.157 (0.12)	0.055 (0.1)	0.101 (0.1)
	Interpersonal & Communication Skills	-0.116 (0.05)	0.005 (0.05)	-0.028 (0.07)	0.236 (0.12)	0.095 (0.1)	0.175 (0.1)
Site 3	Patient Care	-0.185 (0.07)	-0.183 (0.06)	0.206 (0.09)	0.316 (0.08)	0.950 (0.18)	0.839 (0.22)
	Medical Knowledge	-0.349 (0.06)	-0.413 (0.06)	0.390 (0.09)	0.409 (0.08)	1.146 (0.17)	0.906 (0.21)
	Systems- Based Practice	-0.376 (0.07)	-0.402 (0.06)	0.052 (0.09)	0.216 (0.08)	0.783 (0.17)	0.746 (0.21)
	Practice- Based Learning & Improvement	-0.109 (0.07)	-0.131 (0.07)	0.076 (0.1)	0.115 (0.09)	0.550 (0.19)	0.525 (0.23)
	Professionalism	-0.182 (0.07)	-0.120 (0.06)	0.157 (0.09)	0.291 (0.08)	0.762 (0.17)	0.666 (0.21)
	Interpersonal & Communication Skills	-0.092 (0.07)	-0.080 (0.06)	0.261 (0.13)	0.539 (0.11)	0.882 (0.32)	1.102 (0.53)

©2020 Klein R et al. *JAMA Network Open*.

Site 4	Patient Care	-0.072 (0.08)	0.151 (0.1)	NA	NA	0.128 (0.09)	-0.183 (0.1)
	Medical Knowledge	-0.067 (0.08)	0.140 (0.11)	NA	NA	0.185 (0.1)	-0.246 (0.11)
	Systems- Based Practice	-0.096 (0.08)	0.150 (0.1)	NA	NA	0.126 (0.09)	-0.201 (0.11)
	Practice- Based Learning & Improvement	-0.055 (0.08)	0.100 (0.11)	NA	NA	0.073 (0.1)	-0.157 (0.11)
	Professionalism	-0.136 (0.08)	0.222 (0.11)	NA	NA	0.099 (0.1)	-0.150 (0.11
	Interpersonal & Communication Skills	-0.110 (0.09)	0.194 (0.12)	NA	NA	0.152 (0.1)	-0.189 (0.12)
Site 5	Patient Care	-0.517 (0.1)	-0.527 (0.13)	-0.141 (0.15)	0.163 (0.1)	0.914 (0.18)	0.696 (0.14)
	Medical Knowledge	-0.449 (0.11)	-0.539 (0.13)	-0.133 (0.16)	0.115 (0.1)	0.922 (0.18)	0.731 (0.14)
	Systems- Based Practice	-0.441 (0.11)	-0.488 (0.13)	-0.183 (0.16)	0.185 (0.1)	0.837 (0.18)	0.666 (0.14)
	Practice- Based Learning & Improvement	NA	NA	NA	NA	NA	NA
	Professionalism	-0.353 (0.11)	-0.462 (0.14)	-0.172 (0.17)	0.136 (0.11)	0.794 (0.19)	0.584 (0.15)
	Interpersonal & Communication Skills	-0.454 (0.11)	-0.502 (0.14)	-0.025 (0.16)	0.186 (0.11)	0.780 (0.19)	0.579 (0.15)
Site 6	Patient Care	-0.341 (0.07)	-0.414 (0.1)	0.136 (0.08)	-0.158 (0.14)	0.542 (0.08)	0.439 (0.16)
	Medical Knowledge	-0.566 (0.09)	-0.487 (0.14)	0.221 (0.11)	-0.107 (0.2)	0.737 (0.1)	0.759 (0.22)
	Systems- Based Practice	-0.348 (0.08)	-0.245 (0.12)	0.053 (0.09)	-0.155 (0.17)	0.508 (0.09)	0.494 (0.18)
	Practice- Based Learning & Improvement	-0.407 (0.08)	-0.460 (0.13)	0.097 (0.1)	0.008 (0.18)	0.659 (0.1)	0.440 (0.2)
	Professionalism	-0.453 (0.09)	-0.494 (0.14)	0.240 (0.12)	0.008 (0.21)	0.558 (0.11)	0.631 (0.23)
	Interpersonal & Communication Skills	NA	NA	NA	NA	NA	NA

Abbreviations: p=p-value SE=standard error, NA=not assessed
*Adjusted standardized scores for IM Core Competencies as determined by the Accreditation Council for Graduate Medical Education[17]
±Mean and Standard Errors obtained from a random-intercept mixed model adjusted for the clustering of residents within faculty within programs and baseline IM In-Training Examination Percentile Rank

©2020 Klein R et al. *JAMA Network Open*.
